# Injuries of the Head

**Published:** 1857-02

**Authors:** J. T. Jameson

**Affiliations:** South Otselic, Chenango Co., New York


ART. III.—*Injuries of the Head.* By J. T. JAMESON, South Otselic, Chenango Co., New York.

CASE I. A boy æt. 15, fell from the top of a building three stories high, and lighted on some planks beneath. He was insensible for some minutes. He was seen, soon afterward, by myself and partner, Dr. Coates. On examining the head, we found a ragged wound, about two inches long, over the right parietal bone, which was exposed to some extent. On further inspection, it was discovered that a large piece of wood had been driven in an oblique direction through the parietal bone, forming a regular wedge, and so firmly impacted that it was impossible for us to remove it entire. It was, therefore, divided into small portions, and even then, great force was required for their removal. One small portion could not be extracted. Lint, spread with simple cerate, was applied to the wound.

Four hours afterward. Reäction established; patient perfectly sensible; occasional slight twitching of the extremities.

V. S. ℥viij.; cal. grs. iij. every four hours.

Next morning, Oct. 12th. Patient has slept four or five hours; perfectly rational; no pain in the head; skin hot and dry; pulse frequent and sharp; twitchings continue; wound looks well.

V. S. ℥xij.; cont. cal.

13th. Slept well last night; no pain in the head; skin hot; pulse frequent and soft; bowels moved twice; wound suppurating, and looking well; salivation commencing.

17th. Boy has been going on well since last date; the wound looking well, and discharging healthy pus.

Nov. 4th. He complains of slight occasional pain in the temple, and between the upper surface of the globe of the eye and the orbit.

The wound continued open for about four months, when the portion of wood above alluded to was removed, after which it speedily closed.

*Remarks.* Whether the piece of wood was removed as soon as it might have been, I am now unable to express anything certain in reference to, the case having occurred many years ago, and the above constituting all the record which I have of it. I should now suppose that it would have become sufficiently loosened to have admitted of removal at an earlier period. However this may be, the case, I consider, serves to illustrate the advantage of an early and decided antiphlogistic treatment in such cases, with the design of *preventing* inflammation and the fatal result so generally following thereupon.

Case II. A carter, æt. 40, of robust constitution, was knocked down by one of his horses and trod upon. He was stated to have been insensible for a short time, and then got up and walked a few yards to an adjoining house.

When seen about two hours after the accident, he was perfectly rational. There were two considerable scalp wounds, and in one of them, situated near the inferior edge of the parietal bone, there was detected a fracture of some extent, with slight depression. There was much appearance of bruising about the upper part of the throat, as if the horse's foot had been there.

The wounds were dressed with adhesive plaster. V. S. ℥xvj., 16 leeches to the throat. Purgative.

2d day. Pulse about 80, full and jerking; conjunctiva injected.

V. S. ℥xvj.

3d day. V. S. ℥xij, and more leeches to the head and throat.

The wounds looked well, the greater portion having united by the first intention. He was sitting up in the course of five days, and was soon perfectly recovered.

Case III.—James Ryley, æt. 38. I was called to see this man about half after seven, on Wednesday evening, and ascertained that, about fifteen minutes prior to my arrival, he had fallen on his head, on the pavement, from the top of a cart loaded with flour. There was no wound of the scalp, and no fracture to be detected; face pale; eyes closed; pulse of moderate strength; perfectly rational; blood flows rather copiously from the right ear; in the course of a few minutes he vomited freely.

He was placed in bed, and warmth applied to the extremities.

I visited him again in a couple of hours. He is rational, but complains of feeling a great noise in the head; has vomited frequently, and chiefly blood, a gush of blood also taking place from the ear during the act of vomiting; pulse full and sharp.

V. S. ℥viij, when he became faint; calomel, grs. v.

Thursday morning. Has pretty severe pain in the head; pulse bounding and sharp; skin hot; bowels moved once; has vomited a dark colored pitchy matter.

V. S. ℥xij; cal., grs. ij, every three hours.

Friday morning. Pain in the head much relieved; pulse soft and free from jerk; skin cool; gums indicate commencing salivation; there has been a copious discharge of clear fluid from the right ear.

Vespere. Patient still appears in a favorable state, although a little more feverish than in the morning, and the pulse more frequent and full; constant discharge during the day, of clear fluid from the ear. In the course of the evening the patient was got up in order to make his bed. He felt very faint during the operation, and about half an hour afterward, I was sent for, and found him complaining of most agonizing pain in the head, extending from back to front; pulse 50, and oppressed; skin cool.

I endeavored to bleed him from the arm, but could not obtain more than a few ounces, the blood being thick and dark, and flowing very slowly; 10 leeches to the head; empl. lyttæ nuchæ. An anodyne with a view to mitigate the pain somewhat.

Saturday morning. Pain in the head continues about the same; pulse less slow and oppressed.

20 leeches to the head.

Noon. Patient pale, sunken, restless, and with difficulty confined to bed; grinding of the teeth, and retraction of the angles of the mouth; pupils contracted.

Ress. Anodyne, and ordered a turpentine enema with 10 drops of tinct. opii.

He remained much calmer during the afternoon, but toward evening became restless again; pulse small and frequent; pupils contracted; slight trachael râle.

He died in the night.

*Remarks.* In all probability there was, in the preceding case, fracture of the base of the cranium, and extending through the petrous portion of the temporal bone. We are frequently disposed, in unsuccessful cases, to imagine that if something different had been done, or something left undone, the termination might have been different, and in accordance with this popular reasoning, I have always thought that had the mercury been continued in Ryley's case, and above all, had the getting up been avoided, and the recumbent position, with perfect quietude of body and mind, been strictly observed, the result might have been different. I thus judge, because his condition was, on the whole, so favorable up to the period of getting up. I am disposed to consider that internal bleeding took place at the time of getting up, and, although, from this period, the case was probably hopeless under any treatment, yet I should now regard the second application of leeches as having been injurious. The following quotation from Dr. Williams, may not be without interest in connection with the preceding case. The Dr. states "that after injuries of the head, a serous fluid in large quantity may escape from the meatus. It may or may not be preceded by blood. He believes that in all cases where this clear fluid escapes, there is fracture through the labyrinth, and hence arises a question as to whether this fluid is an increased secretion of the liquor cotunnius, whether serum of blood or fluid from the membranes of the brain. It certainly is not the serum of the blood, inasmuch as several ounces have drained away in the course of a few hours; and it has been tested, and was found not to be serum. In one instance it was collected, and considerably exceeded a pint during the night."

Case IV. Charles Messenger, æt. 15. I was sent for about 8, P. M., of the 7th of October, 1853, and was informed that on the 5th, in the evening, the boy was struck by a horse on the right side of the head, immediately over the ear and mastoid process. Being alone in the field, there is only his own narrative to depend upon. He says that he lay insensible, but cannot tell how long. When consciousness returned, he got up and walked to the house, experiencing severe pain in the head. There was a little bleeding from the ear, and considerable swelling over the mastoid process.

6th. He was out of doors, but complained of pain in the head. During the night the pain in the head became so severe, he was unable to sleep, and vomited a couple of worms.

7th. This morning he vomited another worm. He took a dose of oil, which was rejected, and then a dose of salts, which was retained, but have not operated as yet. The pain in the head has been very severe during the day, and especially this afternoon.

Present state. There is a slight wound at the margin of the external meatus of the right ear, and the passage contains some coagulated blood; lividity over the mastoid process, and also a little in front of the ear; paralysis of the right upper eyelid, so that he is unable to open the eye; the pupil of this eye is dilated much more than the other, and does not contract on bringing a lighted candle near the eye; the retina retains its sensibility, and light is painful to the eye; pulse slow, (about 60,) and laboring; frequent spasmodic jerkings of the extremities, and grinding of the teeth; tongue slightly coated; neither head nor surface generally particularly hot; has been a little incoherent this afternoon.

V. S. ℥xij, the blood having an arterial color; empl. lyttæ nuchæ; cal. gr. x, followed by a teaspoonful of salts in six hours; mustard poultices to the feet.

8th. Mane. Much the same; pulse 60, and laboring as last night; paralyses of levator palpebræ continues, but by closing the other eye, he can raise the lid a little; pupil still dilated, but seems a little more sensible to light; spasmodic twitchings and grinding of the teeth rather diminished; blister acted well; the blood, through mistake, has been thrown away, but from the description, I inferred that it was buffed; bowels moved once, but no worms expelled.

V. S. ℥x, when he became faint, and the pulse frequent. Cal. grs. ij, every four hours.

9th. Mane. A little improvement; pulse 72; skin warmer than yesterday; tongue more coated; mouth feels sore, and the gums indicate mercurial influence; can raise the eyelid a very little, and by closing the other eye can half raise it; pupil still dilated, and but little influence by light; pain in the head decidedly less; noise aggravates pain in the head. I do not observe any twitchings or grinding of the teeth this morning; bowels moved slightly several times, the evacuations being of a dark green color; blood not buffed.

Omit cal. Hydr. c. creta, grs. v, every four hours; sod. bicarb.

10th. Feels better; pulse 62, but not so full and laboring; tongue cleaning at the edges; mouth pretty sore, and mercurial fœtor decided; does not feel any pain in the head, except when a noise is made in the house; seems to have a little more power over the eyelid, and the pupil is less dilated; bowels moved two or three times.

Cont. pulv. bis in die.

11th. About the same; a cough, in consequence of a cold which he had prior to the accident, is rather troublesome.

Cont. pulv. and add morph. sulph., gr. $\frac{1}{6}$; pot. hydriod., grs. ij, ter in die. Ol. ricini, mane.

12th. Appears better; tongue cleaner; pulse 76, and soft; has considerable supra orbital pain, arising, most probably, from the cold, which is not so troublesome.

Cont. pulv. each night, and pot. hydriod. ter in die.

14th. Seems convalescent; tongue almost clean; pulse 72; has slept well the last two nights; has pain in the head occasionally, and especially during a noise; paralysis of the lid still continues, and the pupil is still more dilated than the other.

Rep. pulv. a few times, and continue the hydriodate of potash a few days.

I did not see the patient again until the 31st of October, when I accidentally met him in the street. He appeared to be quite well, and free from pain in the head, but could only half elevate the upper lid; the pupil was contracted like the other, but I was considerably surprised, not having observed anything of the kind during my visits, to find the cornea of this eye turned upward and outward, causing double vision. The friends had applied a blister behind the ear, as I had directed at my last visit, in case the eye did not improve. I recommended another to be applied, but the boy feeling so well, they were indisposed to do anything more, and when I saw the patient sometime afterward, he had perfectly recovered from the paralysis and obliquity, the eye being in all respects sound and natural. I have seen him repeatedly since, and found the eye all right.

*Remarks.*—I am not disposed to consider that there was fracture of the base of the skull in the preceding case, but that in consequence of the concussion, a rupture of one or more small vessels, at the base of the brain, was produced, giving rise to a limited extravasation of blood, the pressure of which was particularly experienced by the third pair of nerves. I should suppose that inflammation had commenced at the time of my first visit, and that the boy's life was probably saved by the prompt and decided antiphlogistic treatment adopted. I am unable to afford any satisfactory explanation of the occurrence of the obliquity after the boy became convalescent. As he was confined to bed during my visits, it is barely possible that it might have existed at that time, but having examined the eye so frequently, it seems almost incredible that it should have escaped notice.

Case V. My friend, Dr. Bower, received an order from the coroner to make a post-mortem examination of the body of a boy, æt. 12, who had died after injury received, and he requested me to accompany him. We were informed that, ten days ago, one of his companions had inflicted a blow with a stick on the back part of the boy's head, by which the skull was exposed, and that since the accident, he had complained more or less of pain in the head, but yet was not sufficiently sick to be confined to the house. On the sixth day, a quack was sent for, who dressed the wound with some kind of plaster or salve. He died, in a comatose state, on the tenth day from the reception of the injury.

We found the wound still open, the bone being apparently uninjured. On dissecting back the scalp with a view to the removal of the calvarium, there was observed a small collection of pus a little above the wound, and the bone in this place presented a darker hue than natural. On removing the upper portion of the skull, its internal surface, opposite the seat of injury, was found to be healthy, as was also the external surface of the dura mater; but on removing this membrane, a circular deposit of pus of the size of half a dollar, was observed opposite to the suppuration beneath the scalp. On wiping away the pus, the arachnoid appeared to be destroyed, and the surface of the brain presented an irregular aspect, as if ulcerated. The brain otherwise seemed healthy, the vessels only being considerably congested.

Case VI. Michael Burks, æt. 18. When I first visited this patient, he manifested the ordinary symptoms of continued fever, and such I deemed his case to be. He was bled, but soon fainted. He was purged, and febrifuge mixture ordered. He remained in about the same state for four days, when he complained more particularly of his head, and the conjunctiva was injected.

Head shaved, and cold applied.

On the following day he was much worse; conjunctiva more injected; pupil of the left eye widely dilated; left arm in a state of tonic contraction, the fingers tightly grasping the bedclothes; he was unable to articulate, but put out his tongue when desired. He soon became comatose, and died in the night, being the second day from the appearance of the alarming head symptoms.

After his death, I was informed that some little time prior to being taken sick, Burke had received a severe blow on the head, which divided the scalp to the bone, and that since then, he had complained of pain in the head.

*Remarks.* Although, in the absence of a post-mortem examination, nothing certain can be stated in reference to the preceding case, I cannot but express the opinion that the blow was the *fons et origo mali*, being followed by inflammation, ramollissement of the brain, and may be suppuration. I regard it as probable that irreparable mischief had already been accomplished in the brain at the time of my first visit, yet as no mention is made in my notes of local depletion, blistering, and mercury, I consider that these ought to have been put in practice, if they were not, and especially the last mentioned, which ought to have been given to commencing salivation.

From the preceding cases, as well as from all that I have read and observed, I deduce the practical inference, that in all cases of injury of the head, even if that injury may not at the time appear of much severity, it is of the utmost importance to watch with much solicitude the future progress of the case, and where fracture of the skull has been sustained, or a severe blow inflicted, not to wait for pains in the head and other bad symptoms to manifest themselves, for then the treatment, however skillfully adapted, will probably come too late, but to *anticipate and prevent* inflammation and its destructive consequences, by a decided antiphlogistic treatment, as soon as the patient has recovered from the shock of the accident, and especially the administration of mercury to the securing of its specific influence upon the system, if the character of the symptoms so demand. By the adoption of such a method of treatment, combined with perfect repose of body and mind, I believe we shall put in operation the very best means in our power for securing the well-being of our patient. If I am asked, whether I would abstract blood from every individual experiencing a fracture of the cranium, or on whose head a heavy blow had been inflicted, I reply that I should, either locally or generally, unless there was something in the case palpably to forbid it, and having done this, and moved the bowels, if necessary, I should then commence with small doses of mercury, and be guided by the subsequent progress of the case, as to the repetition of the bleeding, and as to how far the mercurial was carried.

In relation to Case V, it may serve to prove, in addition to the many others that might be adduced, the extreme uncertainty which must attend the use of the trephine under such circumstances.

The following cases were observed during my attendance at the Edinburgh Infirmary, several years ago:

Case VII. A man who had been run over by a coach, was brought into the Infirmary. There was a wound on the back part of the head, and a fracture of a triangular form, with very slight depression. He was in a state of profound coma when I saw him; respiration stertorous; eyes closed; pupils contracted; forehead hot, and surface generally, warm; pulse small and fluttering. He died in a few hours afterward.

*Post-mortem.* On removing the calvarium, a very small coagulum of blood was found immediately beneath the seat of injury. The principal effusion was on the surface of the left hemisphere, the greater portion of which was covered with a thin stratum of blood. In the substance of the anterior lobe of the same side near the surface, laceration had occurred, and an effusion of blood to the extent of about a dollar had taken place. The brain, in other respects appeared to be healthy.

Case VIII. A woman, who the night but one previous had been found lying at the foot of a common stairs, was brought into the Infirmary. She exhibited all the symptoms of compression of the brain: profound coma; puffing respiration; skin warm; pulse small and indistinct; pupils rather dilated; two wounds of the scalp, one anteriorly, and the other posteriorly, but no fracture was discovered. She died about six hours after admission.

*Post-mortem.* Beneath the dura mater, on the left side, and occupying the anterior and lateral part, as also a part of the base of the cranium, was a large coagulum of blood. On making a section of the left anterior lobe, the clot of blood was found extending into its substance to the distance of an inch, thus proving that the source of the hæmorrhage had been a laceration of the brain. The brain, generally, was much congested. From the occipital protruberance, the seat of the posterior wound, a fracture was found to extend to the foramen magnum, and another in a transverse direction in the course of the lateral sinus. There was simply a fissure, and no displacement of bone.

Case IX. A man, æt. 45, who had leaped through a window, and fallen from a height of three stories, was admitted under Syme. He was completely insensible for a quarter of an hour after the accident, and subsequently continued in a semi comatose state, answering questions when roused, but immediately relapsing again into a torpid state. He remained thus for four days, gradually becoming more comatose until his death on the fifth day after the accident. The left arm was in a state of rigidity.

*Post-mort.* No fracture of the cranium could be discovered, nor any abnormal appearance external to the dura mater. On removing this membrane, the whole of the left hemisphere, and extending to the base of the brain, was found covered by a thin stratum of coagulated blood. In the substance of the right hemisphere, two or three clots of blood were found, arising from the laceration of the brain at these particular spots. The substance of the brain, generally, was preternaturally vascular.

Case X. A woman was admitted under Watson, in a state of perfect coma, having on the previous evening fallen down a pair of stairs, and been insensible ever since. There is no wound of the scalp, but only an indistinct puffing in the occipital region; breathing slightly stertorous; face pale; pupil of right eye contracted; skin warm; pulse moderately full; complete relaxation of all the muscles.

The trephine was applied over the occipital base in the situation of the puffing. I noticed a movement of the upper extremities when the scalp was incised. No blood was found beneath the bone, and the wound being closed, the patient was conveyed to bed in statu quo. She died the following day.

*Post-mort.* A large clot of blood was found covering the left anterio lobe, and imbedded in its substance which was softened around the extravasation, and presented an irregular aspect as if ulcerated. Right hemisphere perfectly healthy. There was fracture of the inferior part of the occipital bone, and extending to the base of the cranium.

CASE XI. A woman was admitted into the Infirmary who had fallen from a considerable height on the back of the head, causing a wound of the scalp, but no discoverable fracture or depression. There were all the symptoms of compression with considerable bleeding from the left ear. She died about twelve hours after the accident.

*Post-mort.* The whole of the upper surface of the hemispheres, and particularly over the anterior lobes, was covered with a thin coagulum of blood. Blood was also extravasated in the tuber annulare and cerebellum. There was extensive fracture of the occipital and temporal bones, extending across the course of the lateral sinus, and across the petrous portion of the temporal bone.

CASE XII. A boy, æt. 10, was admitted into the hospital, having been run over by a cart, the wheel of which had passed over the head in its long diameter. There were symptoms of compression with copious hæmorrhage from the right ear, terminating in death after a few hours.

*Post-mort.* The base of the cranium was found fractured completely across, passing through the petrous portion of the right temporal bone. The lesser alæ of the sphenoid bone were thrust upward into the substance of the anterior lobes which was broken down in consequence. There was scarcely any extravasation of blood either in the substance, or upon the surface of the brain.

*Remarks.* The preceding five cases seem calculated to teach us how dubious the use of the trephine must be in such instances, so dubious as, it appears to me, to forbid its application altogether, that is, in cases of compression from injury, but without any discoverable fracture and depression. The chances are so very small that a coagulum will be found beneath the bone at the seat of injury, and not, as in the above cases, beneath the dura mater, and at an opposite quarter to where the blow was received, or the blood be diffused in a thin lamina over an extensive surface, or the substance of the brain itself be lacerated and blood effused there, that the operation, according to my judgment, cannot be recommended. Many such cases are, of course, utterly hopeless under any system of treatment, but if there was a possibility for the patient to reover, I believe that he would realize it by a faithful adherance to the antiphlogistic means already referred to, and especially the use of mercury; and as, probably, the absorption of a coagulum, or the healing of a laceration of the brain, is a work demanding considerable time, we may learn the importance of exercising due perseverance in our treatment, and especially of inculcating suitable repose of body and mind for a sufficiently long period. Case VIII is interesting in relation to these points. I am sorry not to be able to furnish the treatment adopted in that case, but from what the post-mortem revealed, I should consider that there was not sufficient mischief produced, to preclude the possibility, and I think that I should not be going too far to add probability, of recovery under the faithful administration of the antiphlogistic treatment above recommended.

Case XIII. A man was admitted under Syme, with fracture of the cranium. There was a wound of the scalp on the upper and posterior part of the left parietal bone, which was fractured, and a portion of bone depressed. The patient was insensible, and there was paralysis of the right side. Syme made a crucial incision, and having dissected back the flaps, removed a portion of the cranium with the trephine, after which the depressed portion was elevated. The man became sensible immediately after the operation, and answered questions rationally. For some days subsequently, he had pain in the head and febrile symptoms, but by the repeated application of leeches, low diet, and absolute rest, these symptoms subsided, and he recovered the use of his right side. The pulsation of the brain was very distinct for several weeks after the operation, but as the wound filled up with granulations and cicatrization proceeded, it subsided, and is now scarcely perceptible. Three months after the accident, the man was perfectly recovered, and the wound healed.

Case XIV. A man was admitted under Syme, who, in a fit of insanity, had shot himself in the head with a pistol. A portion of the cranium was driven in, which was removed by the trephine, together with the ball. He appeared to be doing well for some days, when the wound assumed a sloughy aspect, and a pulsating tumor protruded from it which Syme removed with the knife, and afterward applied a compress of lint and bandage. The patient did not complain of any pain in the head; pulse frequent; skin hot. The tumor reäppeared on the following day—a firm compress was applied over it. His countenance now began to assume a pale sallow hue, with general prostration, and occasional delirium.

July 17th. The patient appears in a semi-comatose state, and fast sinking. There has not been much protrusion for the last two or three days.

18th. Died in the night.

*Post-mort.* There was an aperture capable of admitting two fingers in the cranium and dura mater, and through which a fungous mass protruded. On making a section of the hemisphere of that side, a large portion of it, extending from the fungus to within the eighth of an inch of the lateral ventricle, was broken down, and in a state of suppuration.

Case XV. Andrew Robb, æt. 17, admitted under Syme. Sixteen days ago, he received a blow on the back part of his head, from a bar of iron. No insensibility followed the injury, although the cranium was fractured, and three pieces of bone, including both tables, removed. Two days after the accident, he was bled from the arm on account of headache and fever, the blood being buffed and cupped. An antiphlogistic regimen has been strictly observed, and the bowels have been regularly moved. No unpleasant symptoms occurred until a few days ago, when the scalp at the seat of injury was observed to project, and, on inspection of the wound, a fungous looking growth having a pulsatory movement, was seen to protrude through it; pulse 120, and rather sharp; tongue perfectly clean; respiration natural; no headache; tranquil expression of countenance; good appetite; no thirst. The fungous mass is about the size of a pigeon's egg. Syme enlarged the wound by a flap, and having completely exposed the growth, removed it at its base with a bistoury. The part removed exhibited the characters of cerebral texture. There was a little bleeding. A portion of lint was placed under the flap, and a bandage over the whole.

Next day, Nov. 6th. Pulse 130, and soft; tongue clean and moist; no complaint; bowels moved this morning.

7th. Pulse 120. In other respects the same as yesterday.

8th. Pulse 120; no complaint; healthy discharge exuding through the dressings. Syme thought it advisable to dress the wound. No appearance of further protrusion, and the exposed brain has a sloughy aspect, which he considered favorable. A portion of dry lint was again placed beneath the scalp, and compresses with bandage as before.

9th. Pulse 116; slight heat of skin; tongue slightly coated; complains of pain in the forehead.

Cal., grs. iij; pulv. antimon., grs. iij, h. s. s.

10th. Appears in all respects better, except that he complains of pain in the forehead, which was relieved by slackening the bandage.

11th. The pain in the head recurred during the night, and continued until ten this morning, when he took a powder of pulv. Doveri, grs. viij, pulv. Jacobi, grs. iij, and since then he has been quite easy; pulse 84, and soft; tongue quite clean.

12th. Pulse 112 and sharp; tongue some coated; had nausea and vomiting last evening; slight pain of the head during the night; feels easy at present, and looks cheerful. The wound, being examined, was found to present an appearance similar to that of the original fungus, only smaller. The projecting part was removed as before, and the same dressings applied. Bowels being imperfectly moved, he was ordered mist. senna co., and enema if necessary.

13th. Bowels freely moved; pulse 104, and softer; tongue clean; slight pain in the head during the night: quite easy at present, and looks calm.

14th. Pulse 112; skin cool; three evacuations after an enema; no pain in the head since yesterday, except slightly for about ten minutes this morning; looks and feels better.

15th. Pulse 108; more pain in the head, and evinces a great aversion to noise, and disturbance of any kind; the dressings were removed, and the fungous growth was found reproduced to the same extent as before, its surface having a distinctly sloughy aspect; dressings reäpplied.

16th. Almost general redness of the skin similar to that of scarlatina; tongue red, smooth, and moist; pulse 112; bowels moved once; makes no complaint; took mist. senna co. this morning.

17th. The redness has disappeared from the limbs, but still remains on the trunk, though very faint; pulse 110; tongue not so red, and moist; says that he feels perfectly well.

18th. The fungous growth has sloughed to its base, and seems nearly detached; pulse 96; tongue as yesterday; slight confusion of ideas and expression which may, perhaps, be the effect of the opiate (tinct. opii, ♏ xxx) taken last night; respiration slow and natural; redness of skin disappeared; bowels moved once.

19th. Has taken no food since yesterday, and has been very thirsty; pain in the head and back; has wandered much in his ideas, and been very restless; pupils dilated; appearance of languor and depression; frequent moaning; pulse 70; two evacuations after enema.

20th. Much incoherency and restlessness during the night, but to-day seems quite rational; occasional moaning and appearance of distress; pulse 80, with slight irregularity; a great part of the slough has separated, and the remainder seems ready to do so; tongue clean and natural; no appetite; no thirst; no complaint but of general uneasiness; poultice to the wound.

22d. Soon after visit on the 20th, he became insensible; constantly moaning; eyes closed; countenance sunken, and of a pale sallow hue; slight convulsive twitchings; never recovered from insensibility, and died last night at seven o'clock.

*Post-mort.* The body was considerably emaciated. On removing the scalp, an irregular aperture, about the size of half a dollar, was seen in the cranium, with the sloughy brain protruding through it. The surface of the brain was observed to be remarkably dry, and near the margin of the fungous growth, the dura mater became adherent to the brain. On cutting into this hemisphere of the brain, it was found to be softened, and reduced to a pulpy mass; a large portion of the posterior lobe, all around the seat of injury, was of a dark color, softened, and almost putrid; this diseased portion communicated with the lateral ventricle in which was found a considerable quantity of a sero-purulent fluid, derived, apparently, from the adjoining putrid cerebral substance. The right hemisphere appeared to be healthy, with the exception of increased vascularity.

*Remarks.* So far as my knowledge extends, true hernia cerebri, as the above cases would be designated, is most commonly fatal, and the condition of the brain, as revealed by post mortem examination, sufficiently accounts for its being so. The nature of the dieease appears to be, inflammation of the cerebral substance, terminating in ramollissement and suppuration. If such is the case, must not our hopes center in *prevention?*. And do we not find in this a confirmation of the propr ety and necessity of carrying out the strict antiphlogistic treatment above recommended in all cases of injury of the head of any severity? I may be referred to the second of the preceding cases as an instance in which this appears to have been done, and yet to no purpose, but, without designing the least reflection on the distinguished surgeon who had charge of this case, I must state that what I understand by antiphlogistic treatment was not carried out in Robb's case. It is true, that two days after the accident, he was once bled from the arm, the blood being buffed and cupped, but we read of no repetition of the bleeding either generally or locally, and, above all, of no administration of mercury until its constitutional influence was obtained, for it is to the prompt and full use of this medicine that I attach great importance. Let me not be understood as insinuating that Robb's case would not have pursued precisely the course it did, even had my idea of the antiphlogistic treatment been carried out, but as simply explaining what I mean by that treatment, and wherein it was defective in the particular case of Robb. I know not that any mode of treatment is adequate to prevent the disease, but from what I understand to be the source of danger in all injuries of the head, and the pathology of the disease under present consideration, namely, inflammation and its results, I should unhesitatingly adopt the antiphlogistic treatment as above explained, and even when the disease had made some progress, I should place more confidence in this treatment, modified to the then condition of the patient, than in any other, that is, local depletion, counter-irritation, and mercury to commencing salivation.

